# Influence of albumin concentration in priming solution on blood viscosity under hypothermic conditions

**Published:** 2009-05

**Authors:** Ismail Sapmaz, Sinasi Manduz, Umut S Sanri, Oguz Karahan, Kasim Dogan

**Affiliations:** Sivas Numune Hastanesi, Sivas, Turkey; Cumhuriyet Universty, Sivas, Turkey; Cumhuriyet Universty, Sivas, Turkey; Cumhuriyet Universty, Sivas, Turkey; Cumhuriyet Universty, Sivas, Turkey

## Abstract

**Objective:**

Albumin is used routinely as a plasma volume expander in cardiopulmonary bypass operations. The effect of two different concentrations of albumin in Ringer’s lactate on blood viscosity was explored in this study.

**Methods:**

Ten healthy volunteers (all male) were included in the study, based on their haematocrit levels (42.6 ± 0.96). Using a heparinised 50-ml syringe, 40 cm^3^ of blood were drawn from the antecubital veins of fasting volunteers. Six ml of blood were haemodiluted with 2 ml of albumin (20%), 2 ml of Ringer’s lactate containing albumin (1.3%), and 2 ml of Ringer’s lactate, in order to simulate cardiopulmonary bypass conditions. Test tubes with the solutions were placed in a 15°C water bath for 25 minutes. Viscosity was measured in the haemodiluted blood samples using an Ostwald viscometer. Relative viscosities of samples were assessed with SPSS software and the ANOVA test.

**Results:**

The mean relative viscosity of Ringer’s lactate was 4.19 (± 0.49), that of Ringer’s lactate with 1.3% albumin was 4.30 (± 0.31), and of 20% albumin was 7.32 (± 0.71). The relative viscosity of Ringer’s lactate and Ringer’s lactate with 1.3% albumin were statistically similar, but that of 20% albumin was higher than the Ringer’s lactate and Ringer’s lactate with 1.3% albumin.

**Conclusion:**

Albumin is used as a plasma volume expander in priming solutions for cardiopulmonary bypass operations, but its effect on blood viscosity depends on the concentration of albumin used.

## Summary

Priming solutions are indispensable in cardiopulmonary bypass, but there is no consensus on which priming solution to use.^1^,^2^ If albumin is chosen, there is no information in the literature on the most appropriate concentration to use with regard to the rheological effects of albumin. This study was designed to determine how the albumin concentration used affects blood viscosity.

A preliminary study was done previously.^3^ Hydroxyethyl starch (HES), gelatin, Ringer’s lactate and albumin (1.3% in Ringer’s lactate) were used as priming solutions and the viscosities were measured with a Ostwald viscometer. Ringer’s lactate and Ringer’s lactate with 1.3% albumin had similar viscosities, and their effect on blood viscosity was lower than that of HES and gelatin.^3^ Because of its lower viscosity, albumin was selected for this study.

## Materials and methods

Ten healthy volunteers (all male) were included in the study. Their haematocrits were 42.6 ± 0.96. Using a heparinised 50-ml syringe, 40 cm^3^ of blood was drawn from the antecubital veins of the fasting volunteers. The haematocrits of the blood samples were measured with the simple capillary tube centrifuge technique. In order to eliminate haematocrit differences among the volunteers and the effect of these differences on viscosity values, volunteers were selected according to their haematocrit levels.

The total adult blood volume is about six litres. In practice, we use a 1 500-ml priming solution in adult operations and add 100 ml of a 20% albumin solution. To simulate a cardiopulmonary bypass, we diluted 6 ml of blood with 2 ml of priming solution. The priming solutions consisted of a 20% albumin solution, a 1.3% albumin solution, or Ringer’s lactate. The 1.3% albumin solution was made up of 32 ml of a 20% albumin solution added to 500 ml of Ringer’s lactate.

Test tubes of the solutions were placed in a water bath at 15°C for 25 minutes. The viscosity of the haemodiluted blood samples was measured with an Ostwald viscometer. Fibrinogen and total protein levels of the volunteers’ blood were also measured.

Relative viscosities of the samples were analysed with SPSS software and ANOVA test.

## Results

The mean relative viscosity of Ringer’s lactate was 4.19 (± 0.49), that of Ringer’s lactate with 1.3% albumin was 4.30 (± 0.31), and the relative viscosity of 20% albumin was 7.32 (± 0.71). The viscosity of Ringer’s lactate and Ringer’s lactate with 1.3% albumin were not significantly different. The relative viscosity of 20% albumin was higher than that of Ringer’s lactate and Ringer’s lactate with 1.3% albumin. Fibrinogen and total protein levels were within the normal range.

## Discussion

There is no consensus on the most appropriate albumin concentration for cardiopulmonary bypass priming solutions. Choices in albumin concentration vary from 25%^1^,^4^ to 4%,^5^ and we used a 1.3% and 20% albumin solution. The literature shows the highest concentration of albumin used as a priming solution was 25%. We used a 20% albumin solution because this is the commercial packaging available in Turkey.

The effect of priming solutions on blood viscosity is important. Blood flow is determined by several factors, including viscosity.^6^ Under hypothermic conditions, viscosity of haemodiluted blood is very high^3^ and this is of critical importance. Haematocrit is a major determinant of blood viscosity.^7^ In order to eliminate haematocrit differences between volunteers, and the influence of these differences on the blood viscosity, volunteers with similar hematocrit values were selected for the study.

Iin the literature, the limit of deep hypothermia is generally accepted as 15°C.^8^,^9^ We tried to simulate the most extreme conditions by exposing the blood samples to 15°C. The influence of high concentrations of albumin (20%) on blood viscosity was not surprising because of the high molecular weight of albumin, and our previous study^3^ had shown that although gelatine and albumin have similar molecular weights, albumin had a lower viscosity than gelatin and HES.

## Conclusion

By changing the albumin concentration of the priming solution, blood viscosity is changed while keeping the haematocrit constant. A high albumin concentration (20%) increases the blood viscosity at 15°C when compared to Ringer’s lactate and a 1.3% albumin solution. Hypoalbuminaemia causes high blood viscosity by increasing red cell lysophosphatidylcholine.^10^ The ideal albumin concentration for priming solutions still needs to be determined from outcome-based studies.
